# Seminal Plasma Proteins Associated with The Fertility of Brahman Bulls in The Colombian Low Tropics

**DOI:** 10.21315/tlsr2023.34.3.14

**Published:** 2023-09-30

**Authors:** Liliana J. Chacón, Germán D. Yepes, Jaime Cardozo, Fabian Rueda, Viviana Castillo, Andrés Torres, Jorge Martins, Ariosto Ardila

**Affiliations:** 1 Faculty of Agricultural Science, University of La Salle, Bogota, Colombia; 2 Colombian Agricultural Research Corporation (AGROSAVIA), Tropical Reproduction Group. Kilometer 14 Via Bogotá-Mosquera, Cundinamarca, Colombia; 3 Centre for Agricultural Sciences and Biodiversity, Federal University of Cariri, Rua Icaro de Sousa Moreira, 126, Crato, Ceará, Brazil

**Keywords:** Electrophoresis, Reproduction, Season, Seminal Plasma, Zebu

## Abstract

The sperm interacts with seminal plasma proteins during its transport through the female reproductive tract to reach the oocyte. Seminal plasma proteins have been associated as biomarkers of fertility in bovine males, while two-dimensional electrophoresis in polyacrylamide gels under denaturing conditions (2D-PAGE) is a useful technique for their separation, allowing their subsequent analysis with the aid of specialised software. Brahman bulls are known for their tolerance to tropical conditions such as low-quality pastures, high temperatures, and relative humidity as well as moderate resistance to infestations by parasites and insects. The present study describes the two-dimensional electrophoretic profiles of the seminal plasma proteins in the rainy and dry seasons, associating them with the fertility of Brahman bulls in the Colombian Orinoquía in a 90-days breeding season and a single-sire mating system (1 bull per 50 Brahman cows) with 60 consecutive days of rest. The fertility-related seminal plasma protein spots increased in the dry season. Likewise, a meaningful relationship was found between the protein spots that possibly coincide with the Binder of Sperm Proteins. It was also found that bulls with the highest percentages of pregnancy also had similarities in their 2D seminal plasma maps. We conclude that the seminal plasma protein profile of Brahman bulls raised in the Colombian low tropic changes between rainy and dry seasons, and such changes may influence the reproductive performance of those animals.

**HIGHLIGHTS**

- This is the first description of seminal plasma proteins profile of adult Brahman bulls raised in Colombian low tropics.
- Seminal plasma protein profiles change between rainy and dry seasons in the Colombian low tropic, with some proteins more expressed during the rainy season and other more expressed during the dry season.
- Seminal plasma protein profiles vary from high and low fertility Brahman bulls, as shown by the association between the seminal plasma proteins and the pregnancy rates of bulls.

## INTRODUCTION

Bull fertility is one of the factors that affects the livestock production. The fertility of bulls is responsible for almost 80% of the reproductive capacity of the beef cattle herds (Flowers 2013). Thus, one strategy to improve fertility rates is the use of bulls with the capacity to breed a higher number of females in the shortest time possible. However, bull fertility is not correctly assessed in extensive productive systems (Sellem *et al*. 2015; Rodríguez-Martínez 2003). The breeding soundness evaluation (BSE) is a classic method for estimating the reproductive efficiency of bulls, which provides many benefits to the meat production system before mating (Menegassi *et al*. 2011). However, the BSE and the methods for evaluating the viability of a semen sample do not always accurately predict variations in the fertility of the male (Amann 1989) as the use of additional tools is necessary to enhance the prediction of their fertility.

In this context, the study of the proteomics of seminal plasma constituted a diagnostic method with a sensitive basis to evaluate the fertility of a seminal sample (D’Amours *et al*. 2010; Somashekar *et al*. 2015). Proteomics evaluates the presence and concentration of proteins in seminal plasma samples and establishes associations with sperm function and fertility (Moura, Chapman, *et al*. 2006). For instance, the Binder Sperm Proteins (BSPs) family have been widely related to several sperm functions, such as sperm capacitation, oviductal reservoir formation, and sperm protection (Rego *et al*. 2014; Manjunath & Therien 2002; Magalhães *et al*. 2016). These roles have also been described in other proteins such as the Spermadhesins family, whose participation in sperm motility and the protection mechanism against oxidative damage have been acknowledged (Bustamante Filho 2010; Moura *et al*. 2007).

Brahman bulls (*Bos taurus indicus*) have been developed to withstand higher temperatures than *Bos taurus taurus* bulls (Federación Colombiana de Ganaderos [FEDEGAN] ), and their testicular morphology positively influence the thermoregulatory capability, semen quality and sperm production in bulls (Brito *et al*. 2004). However, there is very little information about their reproductive performance under the climatic conditions of the Colombian Orinoquía, which, in turn, is characterised by high temperatures and relative humidity throughout the year. Information of variation in the seminal plasma protein profiles of those animals due to climatic changes throughout the year, as well as its influence on the pregnancy rates of Zebu cattle in that region is even more scarce. Studies in Brazil reveal that the seminal plasma or sperm membrane protein profiles of small ruminants may change according the rainy and dry seasons in the Brazilian Northeast (van Tilburg *et al*. 2014) or with the increase in the testicular temperature (Rocha *et al*. 2015) but no information of possible variations in the seminal protein profiles of Brahman bulls in the Colombian low tropics is reported. Thus, the objective of this research was to establish the relationship between the variations in the seminal plasma protein profiles of Brahman bulls in different seasons with the parameters of the BSE and the pregnancy rates of the Brahman bulls in the department of Meta, Colombia.

## MATERIALS AND METHODS

All procedures involving animals and their management were approved by the bioethics committee of the Faculty of Agricultural and Animal Sciences in the University of La Salle, Bogotá, Colombia under protocol number 156 of 1 April 2016.

### Location, Climate Registration and Temperature-Humidity Index (THI)

This study was developed in three different cattle farms from the Meta department in the Colombian Orinoquía (Table 1), ranging between 200 and 300 m above sea level in a tropical monsoon climate.

**Figure 1:**
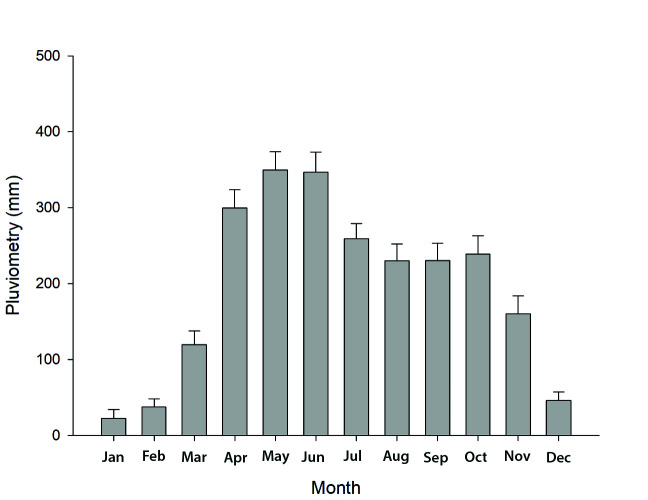
Historical monthly average pluviometry in Meta Department, Colombia. (Raw data obtained by IDEAM, Meta).

The air temperature, relative humidity, and pluviometry data from April 2016 to January 2017 were obtained from the Environmental Research and Hydrology Institute (IDEAM, Meta). The historical pluviometry data from January 2001 to December 2015 were also obtained by the IDEAM to estimate the monthly averages of rain fall and define the rainy and dry seasons of the studied region (Fig. 1).

The temperature-humidity index (THI) was estimated by the following equation described by Valtorta *et al*. (1996):


(1) $$\text{THI}=(1.8\;{\text{T}}^{{}^{\circ}}+32)-(0.55-0.55\;\text{Rh}/100)\;(1.8{\text{T}}^{{}^{\circ}}-26)$$

Where, T° = Air temperature (°Celsius), and Rh = Relative humidity of the air (%).

### Experimental Animals, Definition of Breeding Seasons, Breeding Soundness Evaluation and Bull Pregnancy Rates

Nine adult (5–8 years old) Brahman bulls, in good health and reproductively sound with known reproductive history and presenting a scrotal perimeter higher than 36 cm, were used in the present study. Bulls were extensively managed and fed mainly with Brachiarias with no supplementation.

**Figure 2:**
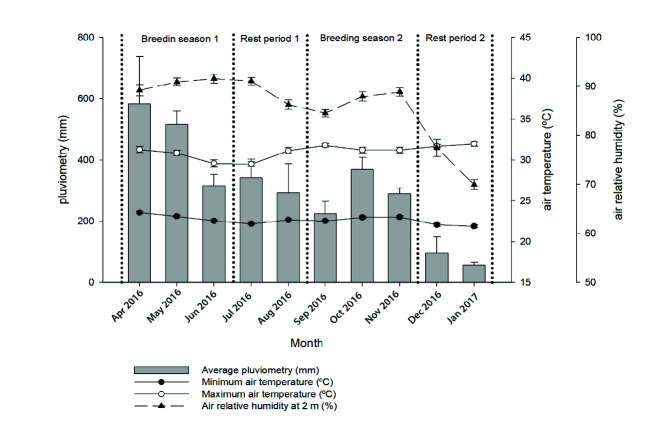
Strategic definition of the breeding and resting seasons in relation to the climatic traits of pluviometry (mm), maximum and minimum air temperatures (°C), and the relative humidity of the air (%) during the year in Orinoquia. Dotted vertical lines represent the moments of breeding soundness evaluations.

Bulls were subjected to a 90-day breeding season that took place during the most rainy months (from April to June 2016) in Orinoquia, followed by a 60-day resting period (July and August 2016). Afterwards, a new 90-day breeding season took place from September to November 2016, followed by an additional 60-day resting period from December 2016 to January 2017. Fig. 2 illustrates the strategic definition of the breeding and resting seasons in relation to the climatic traits of pluviometry (mm), minimum and maximum air temperatures (°C), and the relative humidity of the air (%) of Orinoquia during the year. The breeding was performed using the single sire mating system with a bull:cow ratio of 1:50 as in the model of Fonseca *et al*. (2000).

The breeding soundness evaluation of the bulls was performed at the beginning and the end of each breeding season (dotted vertical lines in Fig. 2) following the International Theriogenology Society parameters (Chenoweth *et al*. 1992). Semen samples were collected by electroejaculation (Ideal^®^ ElectroJac^®^6, Neogen Animal Safety, Lexington, KY), and the sperm analysis was performed in field conditions according to the Brazilian College of Animal Reproduction (CBRA 2013). Briefly, ejaculates were macroscopically evaluated regarding their volume (mL), aspect (watery, serous, milky or creamy), and colour (white, ivory, grey and yellow). To assess the sperm mass motility (0–5), one drop of raw semen from each ejaculate was placed upon a clean and pre-heated (37°C) glass slide without a coverslip and the evaluation was performed using light microscopy (100×). The sperm motility (%) and sperm vigor (0–5) were evaluated by placing one drop of semen upon a clean and pre-heated glass slide (37°C), covered with a coverslip, and evaluated in light microscopy (100–400×). Sperm morphology was evaluated by estimating the percentage of normal cells and the major and minor defects (Merkies *et al*. 2000). One drop of semen was mixed with an eosin-nigrosin stain, smeared upon a clean glass slide, and allowed to air-dry at room temperature. Then, slides were evaluated using light microscopy (1000×). Sperm concentration was determined by the spectrophotometric method (Spermacue, Minitube^®^) by measuring the transmittance at 620 nm of semen samples previously diluted 1:100 with 2.9% sodium citrate. The remaining semen samples were used to obtain the seminal plasma for the study of proteins, as described ahead.

Forty-five days after the end of each breeding season and the removal of the males from the mating group, the females were subjected to the pregnancy detection by rectal palpation and transrectal ultrasonographic examination (6.5 to 11.0 MHz linear transrectal probe, Imago IMV, United Kingdom). Data from the of the pregnancy detection in the females were used to estimate the pregnancy rates (PR) for each bull according to the following formula):


(2) PR=(number of pregnant cows/numbers of served cows)×100

Based on their respective pregnancy percentages, bulls were classified as low fertility (≤ 70%), normal fertility (71%–79%), and high fertility (≥ 80%) (Roncoletta *et al*. 2006).

### Seminal Plasma Collection and Quantification of Seminal Plasma Proteins

Semen samples collected for the BSE were also used to obtain seminal plasma for the study of seminal plasma proteins. Briefly, the semen samples were centrifuged for 5 min at 5,400 x*g*, 4°C, and the resultant supernatant (seminal plasma) was transferred to new tubes. A volume of 1.0 μL/mL of a 5 μM Phenyl Methyl Sulfonyl Fluoride (PMSF) solution was added to the supernatant to avoid proteolysis. Then, samples were transported in liquid nitrogen to the Proteomics area from the Genetics and Molecular Biology laboratory in Agrosavia (Mosquera, Cundinamarca), where these samples again underwent centrifugation under the same conditions, filtered employing 0.22 μm filtering devices and stored at −80ºC until further analysis.

Protein concentration in seminal plasma samples was quantified by the colorimetric method described by (Bradford 1976), employing BSA as standard in different concentrations (50 μg/mL to 800 μg/mL) prior to the two-dimensional electrophoresis procedures.

### Two-Dimensional Electrophoretic Profiles of Seminal Plasma Proteins

The two-dimensional electrophoresis in polyacrylamide gels (2D-PAGE) was performed according to the model of O’Farrell (1975) with modifications. Briefly, 90 μg of seminal plasma proteins were added to a rehydration buffer (8 M urea, 4% CHAPS, 50 mM dithiothreitol (DTT), and 2% ampholytes; Bio-Rad, California, USA) and then incubated with 3–10 immobilised pH gradient strips (Bio-Rad, California, USA), which were passively hydrated overnight (Görg & Weiss 2006) Isoelectric focusing (IEF) was performed through a linear voltage increase up to 250 volts/hour, followed by a rapid voltage increase to 8,200 volts/hour using the Bio-Rad PROTEAN IEF device. Focused strips were sequentially equilibrated by incubation with two different buffers. The first one contained 6 M Urea, 0.375 M Tris (pH 8.8), 2% SDS, 20% glycerol, and 2% dithiothreitol for 15 min. After that, strips were further equilibrated in a similar buffer, changing DTT by 2.5% Iodoacetamide for 15 min. The molecular weight separation was performed in a Mini-PROTEAN^®^ Tetra Cell chamber. Equilibrated strips were carefully placed on the top of 15% polyacrylamide gels, and separation was carried out at 85 V for 3 h. Gels were stained with a Coomassie Brilliant Blue solution and distained with acetic acid-methanol (1:3) solution. Images were acquired in a Gel-Doc XR analyser (Bio-Rad, California, USA), and the spots pattern was analysed by the PD Quest software (Bio-Rad, California, USA; Arora *et al*. 2005).

### Statistical Analysis

THI and other parameters such as the scrotal perimeter, seminal quality parameters, and pregnancy rates were compared in the two different climate periods (rainy and dry) using an ANOVA and a post-hoc mean comparison test (Tukey). In addition, a Pearson correlation test was used to establish associations between the temperature-humidity index, scrotal perimeter, seminal quality parameters, pregnancy percentages, and the relative quantity of each protein spot. Also, the intensities of seminal plasma proteins were compared in high and low fetility bulls with Tukey’s test.

## RESULTS

The averages of the THI values during the breeding seasons and the rest periods are in Table 2, which shows no differences between the rainy and dry seasons (*p* > 0.05). On the other hand, Table 3 shows the overall averages and standard deviations of the scrotal perimeter, seminal parameters and pregnancy rates of all nine studies sires in all breeding soundness examinations. In addition, the classification of bulls is shown according to their fertility. Regarding the parameters of the breeding soundness evaluation, no significant differences were found. However, the pregnancy rates did show significant differences (*p* < 0.05), thus, the general pregnancy rates, in this study, it was 64.0 ± 19.9%. The scrotal circumferences, seminal traits, and the pregnancy rates did not differ among breeding soundness evaluations (Table 4), neither did the pregnancy rates between Breeding Seasons 1 and 2 (64.4 ± 20.6% and 57.6 ± 34.6%, respectively).

**Figure 3:**
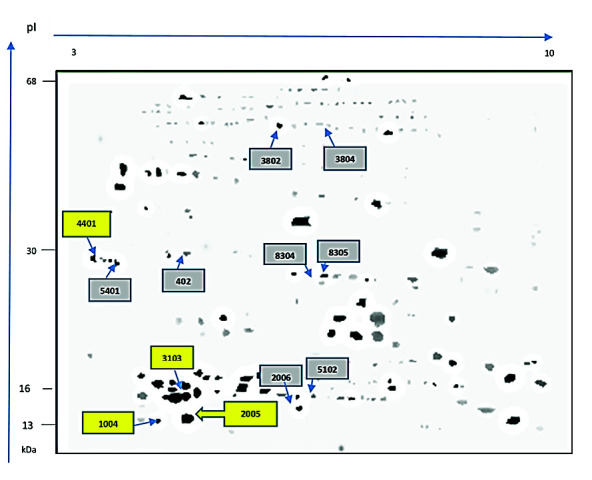
Virtual map generated by the PD-Quest software (Bio-Rad). In yellow we highlight all SPP that had a presentation ≥ 70% in all the samples analysed, in the boxes with the numbers corresponding to the code (SSP) assigned by the software. Spots 2005 (14.1kDa – pl 4.9), 3103 (16.0kDa – pl 4.9, and 1004 (14.0kDa – pl 4.8) could correspond to BSP1 /BSP3, while Spots 4401 (28.6kDa – pl 3.5) would correspond to BSP5. The squares in gray correspond to the spots found in bulls with high or low fertility, with presentation ≥ 70%, referred to in Table 5. The weight of the molecular marker located on the left side of the figure and the value of the isoelectric point at the top are approximate.

The analysis of the gels showed a total of 128 protein spots with molecular weights between 13.4 kDa and 68.2 kDa and isoelectric points (pIs) between 3.5 and 9.7. Not all spots were evidenced in all animals. Fig. 3 shows the virtual map generated by the PD-Quest Software, indicating the SPP that were present in more than 70% of all analysed samples. We found a correlation between the temperature-humidity index and the intensity of Spots 2005 (14.1kDa – pl 4.9) (−0.73; *p* < 0.05) and 3103 (16.0kDa –pl 4.9) (−0.83; *p* < 0.05). Spots number refers to those depicted in Fig. 3. Table 5 shows the changes in the relative concentration (μg) of the proteins because of periods of rain (Breeding Season 1 and Rest Period 1) and dryness (Breeding Season 2 and Rest Period 2), and those that only occurred during the rainy or dry season. An inverse relationship was found between sperm viability and Spot 4401 (28.6kDa – pI 3.5, *r* = − 0.73; *p* < 0.05).

**Figure 4:**
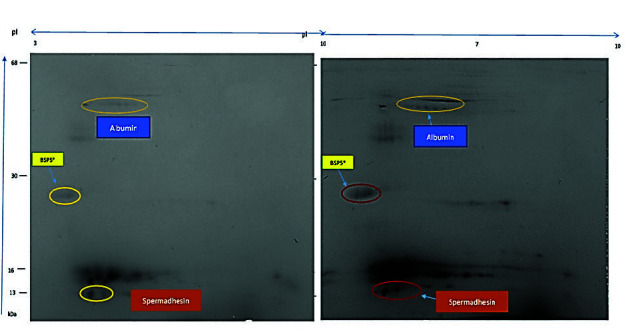
2D electrophoregram of Bull 9 (left) and Bull 7 (right) during mating period in the dry season. * Possibly correspond with these proteins. The weight of the molecular marker located on the left side of the figure and the value of the isoelectric point at the top are approximate.

**Figure 5:**
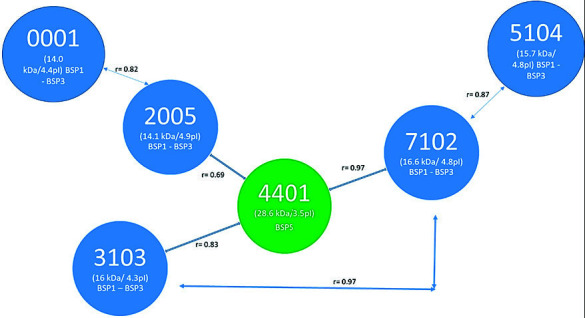
Diagram of relationships (estimated by Pearson’s correlation coefficient) of the intensities of differentially expressed seminal plasma spots of adult Brahman bulls during rainy and dry seasons in Orinoquía, Colombia.

Finally, those bulls with high and low fertility were chosen to establish the association between the SPP and the pregnancy percentage. Table 6 shows the concentrations of SPP whose Mr and PI features correspond to those proteins that have been related to fertility in previous works (Killian *et al*. 1993; Moura, Koc, *et al*. 2006). These relations between SSP concentration and fertility were also determined in separate ways for the rainy and dry seasons (Tables 7 and 8, respectively). Fig. 4 shows the two-dimensional maps of the SPP in the mating period of Bull 9 (high fertility) and Bull 7 (low fertility), showing the location of the protein spots that possibly correspond to Spermadhesins, BSP5, and albumin. Fig. 5 shows the relationships between Protein Spots 0001, 2005, 4401, 3103, 7102 and 5104.

## DISCUSSION

To the best of our knowledge, this is an important study associating seminal plasma proteins with andrological parameters, pregnancy rates of Brahman bulls and climate in Colombian field conditions.

Different researchers have described the risk of moderate to severe heat stress when the temperature-humidity index (THI) is ≥ 76 for bovines (Hammond *et al*. 1998; Bohmanova *et al*. 2007; Lees *et al*. 2018). The animals in the present study remained in areas with mean THI ≥ 76, placing them at the critical limit of production and reproductive performance (Azevedo *et al*. 2005). However, the Brahman is a tropically adapted breed, very robust and resistant to tropical conditions such as low-quality pasture, high parasites incidences, and hot and humid weather (Franke 1980; Façanha *et al*. 2019; Yang *et al*. 2022). Although the animals in the present study were raised in farms where the THI is over 76 the majority of the year (Table 1), they seem to be very adapted do those conditions, since their reproductive characteristic did not differ among evaluations performed in different periods. Moreover, their pregnancy rates were similar in both the rainy and dry seasons.

Although bulls in this experiment were similar in the breeding soundness evaluation, they showed different pregnancy rates (Table 2). This result could reflect the need to deepen the diagnosis of bulls and not only limit themselves to the BSE, introducing molecular analysis of seminal fluid, like proteomic analysis, in order to choose better bulls (Amann 1989; Somashekar *et al*. 2015).

For the first time in Colombia, the 2D electrophoretic profiles of the SPP of Brahman bulls (*Bos taurus indicus*) in reproductive breeding and resting periods were obtained in the department of Meta. The definitions that are indicated in this study as SPP are similar in molecular weight and isoelectric point to that reported by some researchers as seminal plasma proteins (Mortarino *et al*. 1998). For the definitive identification of the SPP it would be necessary to analyze them by mass spectrometry, which was not possible in the present study.

Inverse relationships were found between the THI and Spots 2005 (14.1kDa – pI 4.9) and 3103 (16.0kDa – pI 4.9), spots that coincide with BSP1–BSP3 (Manjunath & Therien 2002). These spots were related to Spot 4401 (28.6kDa – pI 3.5) that coincide with the BSP5, establishing that the possible increases in the THI could cause changes in the concentration of the BSP family. Maybe changes in the concentration of the BSP family cause an alteration in the flow of cholesterol in the sperm membrane, sperm capacitation, and sperm-oocyte interaction, which would negatively impact the reproductive performance. Nevertheless, these findings seem to be of less importance to the pregnancy rates of the present study, since variations in the THI in the rainy and dry season were not significantly different, which is also true of the pregnancy rates of bulls in breeding Seasons 1 and 2.

When comparing the results regarding the fertility ranks of the bulls, we observed that Protein Spots 2006 and 5102, whose molecular weights and isoelectric points (13 to 16 kDa/pI 5.5 to 6.7) are in agreement with those of Spermadhesin Z13, were more concentrated in the less fertile bulls seminal plasma, which would coincide with that mentioned by Killian *et al*. (1993) and Moura, Koc, *et al*. (2006). The fact that Spermadhesin Z13 is present in higher concentrations in bulls with lower pregnancy rates suggests that this protein as an “anti-fertility” factor. It has been demonstrated that high concentrations of Spermadhesin have an inverse association with vigor and sperm motility (Menezes *et al*. 2017) therefore affecting the sperm-oocyte union. This may be the reason for the lower percentage of pregnancy found in bulls with a higher concentration of this protein, as found in this research.

Likewise, Protein Spots 8304 and 8305 (26kDa/pI 6.2), whose molecular weights and pI agree with those of Prostaglandin D synthase type lipocalin (PGDS), showed higher concentrations in the bulls that showed higher pregnancy percentages. This finding coincides with that reported by Killian *et al*. (1993), who established increases in the concentration of lipocalin-type PGD synthase in high fertility bulls and pointed to this protein being associated with fertility (FAP). PGD synthase type lipocalin has been associated with the development and sperm maturation (Gerena *et al*. 1998).

In this research, Bulls 3 and 9 were those with the highest fertility, while Bulls 7 and 8 were those with the lowest fertility. Another finding of this investigation, which is related to bulls of lower fertility (Bulls 7 and 8), is that they showed high concentrations of protein Spots 5401, 402 and 505 (28 to 30kDa/pI 3.6 to 5.2), spots whose molecular weights and pI coincide with BSP5, an acid glycoprotein involved in sperm capacitation, in conjunction with glycosaminoglycans, modulating cholesterol output, which is dependent on time and concentration (Calvete *et al*. 1996). The increase in the concentration of BSP5 has been related to sperm defects and may decrease spermatic viability and affect male fertility (Menezes *et al*. 2017).

Likewise, it was also found that Lower Fertility Bulls 7 and 8 presented higher concentrations of Spots 3802 and 3804 (63 to 64kDa/pI 5.7 to 6.5), which could be albumin, a protein that is produced in the testis, epididymis, and prostate, in addition to the albumin that comes from blood circulation, and which is associated with the alteration of the lipid composition of the plasma membrane, intervening in the training or decapacitation of the sperm. Albumin has also been negatively related to progressive motility and sperm viability (Collodel *et al*. 2019). Likewise, a high concentration of seminal albumin has been negatively related to plasma levels of FSH (Elzanaty *et al*. 2007). FSH has been associated with the proliferation rate of Sertoli cells in the seminiferous tubules, with which sperm production could decrease with increasing albumin (Meroni *et al*. 2002). This could be another reason for the low fertility found in Bulls 7 and 8.

When relating fertility with SPP in the rainy season, the presence of Spot 4401 (28.6 kDa/pI 3.5) was found in the lower fertility bulls, a spot that, as mentioned above, may be BSP5. This protein is not found in the most fertile bulls in this climate season. During the dry season, the seminal plasma of the less fertile bulls, presented Spots 2006 (14.8 kDa/pI 5.4), 5102 (15.6 kDa/pI 6.4), 0002 (15.5 kDa/pI 6.6), 5105 (15.7 kDa/pI 4.9) and 1104 (15.7 kDa/pI 5.5), whose molecular weights and pIs coincide with BSP1/BSP3.

## CONCLUSION

Although the breeding soundness evaluation was conducted before the mating periods, and received a satisfactory qualification, bulls showed differences in pregnancy rates and seminal plasma protein profiles. That suggested a potential association between SPP and the fertility of Brahman bulls raised in different situations in Colombia. In this sense, those bulls with better fertility had higher concentrations of Spots 8304 and 8305. Conversely, Spots 2006, 5102, 5401, 402, 505, 3802 and 3804 were more expressed in those bulls with low fertility. We also conclude that the climatic variations through the year in the Colombian low tropics did not affect reproductive efficiency of Brahman bulls, indicating high adaptability in those animals. These results could serve as a reference to continue conducting studies that allow the selection of the potential sires, using the bovine seminal plasma proteins aiming at a higher reproductive performance for Bos indicus bulls raised under Colombian tropical conditions.

## Figures and Tables

**Table 1:** Coordinates of the three farms in Meta, Colombia used in the present study.

Farm	Department	City	Coordinates
1	Meta		3°29′10″N–73°10′54″W
2	Meta		4°33′27″N–73°20′60′W′
3	Meta		4°17′50″N–72°54′70″W

**Table 2:** Temperature-humidity indexes (mean ± standard deviation) per farm during the 90 days of breeding seasons and the 60 days of resting periods. Breeding and rest periods refer to those depicted in Fig. 2.

Farm	Total	Breeding season 1	Rest period 1	Breeding season 2	Rest period 2
1	76.9 ± 0.5	75.9 ± 0.8	76.2 ± 0.0	77.5 ± 1.1	78.0 ± 1.2
2	77.7 ± 0.0	77.4 ± 0.3	77.3 ± 0.5	78.2 ± 0.3	77.8 ± 0.4
3	77.9 ± 0.6	78.0 ± 1.1	77.2 ± 1.6	78.3 ± 0.4	78.3 ± 0.4

**Table 3:** Mean ± standard deviation of scrotal perimeter, seminal parameters, and pregnancy rates of adult Brahman bulls raised at the Colombian Orinoquía.

Bull ID	SP (cm)	Vol (mL)	Conc (× 10^6^/mL)	MM (1–5)	Mot (%)*	Vig (0–5)	Norm (%)	PR (%)	Fertility
1	38.3 ± 9.2	6.3 ± 1.9	597.5 ± 381.8	3.9 ± 0.5	81.3 ± 2.5*	5.0 ± 0.0	83.8 ± 2.5**	64.0	Low
2	41.0 ± 0.9	5.3 ± 3.6	480.0 ± 408.2	2.3 ± 1.5*	51.3 ± 39.7	4.0 ± 0.8	50.0 ± 35.6	51.0	Low
3	45.4 ± 0.9	6.4 ± 1.1	624.5 ± 358.8	3.5 ± 2.4	67.5 ± 31.8	4.0 ± 2.0	75.0 ± 23.5*	86.0	High
4	41.9 ± 0.8	4.9 ± 0.9	738.3 ± 630.2	4.0 ± 1.2	87.5 ± 2.9**	5.0 ± 0.0	87.8 ± 4.5**	79.0*	Normal
5	43.4 ± 0.9	7.8 ± 1.0*	339.0 ± 313.9	3.5 ± 1.7	80.0 ± 7.1*	4.8 ± 0.5	82.5 ± 8.7*	71.0	Normal
6	40.0 ± 1.7	5.3 ± 2.0	871.3 ± 417.9*	3.5 ± 1.9	73.9 ± 16.0	4.8 ± 0.5	75.0 ± 16.8*	75.0	Normal
7	42.6 ± 0.8	5.9 ± 2.5	609.3 ± 404.0	3.8 ± 2.5	63.8 ± 42.7	3.8 ± 2.5	65.0 ± 43.4	16.0**	Low
8	39.8 ± 0.9	7.8 ± 2.1*	1017.0 ± 647.3**	3.5 ± 1.0	67.5 ± 20.2	4.3 ± 1.0	77.5 ± 8.7	42.0	Low
9	40.1 ± 0.6	8.9 ± 1.3*	568.0 ± 589.3	3.0 ± 2.2	58.8 ± 39.2	3.8 ± 2.5	60.0 ± 40.2	80.0**	High

*Notes*: SP = scrotal perimeter; Vol = seminal volume; Conc = sperm concentration; MM = mass motility; Mot =sperm motility; Vig = sperm vigor; Norm = morphologically normal sperm; PR = pregnancy rates. Significant differences:

* *p* < 0.05;

** *p* < 0.001.

**Table 4:** Means ± standard deviations of scrotal perimeter and seminal traits of Brahman bulls subjected to breeding soundness evaluations in different periods of the year in Orinoquia, Colombia. BSE 1 to 5 refers to those depicted in Fig. 2.

Variable	BSE1	BSE2	BSE3	BSE4	BSE5
Scrotal perimeter (cm)	41.00 ± 1.87	41.33 ± 2.03	41.21 ± 2.66	41.28 ± 2.43	41.72 ± 2.05
Seminal volume (mL)	5.50 ± 2.03	6.61 ± 2.70	6.72 ± 2.24	5.78 ± 2.35	6.78 ± 1.54
Sperm concentration (× 10^6^/mL)	650.67 ± 404.83	578.33 ± 462.22	850.00 ± 239.79	505.44 ± 548.93	628.33 ± 571.08
Sperm progressive motility (%)	81.11 ± 8.21	70.56 ± 30.56	72.22 ± 16.98	65.00 ± 32.02	72.78 ± 27.40
Sperm mass motility (0–5)	3.78 ± 1.20	3.33 ± 1.66	3.89 ± 1.36	3.00 ± 2.00	3.44 ± 1.59
Sperm vigor (0–5)	4.67 ± 0.71	4.56 ± 0.88	4.78 ± 0.441	3.78 ± 1.922	4.33 ± 1.66
Sperm viability (%)	81.67 ± 9.01	76.11 ± 29.25	75.00 ± 15.41	66.67 ± 29.05	74.00 ± 27.84

**Table 5:** Means ± standard deviations of seminal plasma protein concentrations in ejaculates of Brahman bull raised in the Colombian Orinoquía during the rainy and dry seasons.

Protein spot number	MW^*^	pI^*^	Concentration (μg)	

			Rainy season	Dry season
1004	14.0	4.8	22.0 ± 4.4^a^	12.7 ± 3.8^b^
0001	14.0	4.4		3.1 ± 0.8
2005(BSP1)	14.1	4.9	16.4 ± 2.3^a^	6.0 ± 1.3^b^
5104	15.6	4.8		9.9 ± 3.5
5105	15.7	4.9		10.1 ± 2.2
102	15.8	5.3		9.5 ± 3.5
3103(BSP3)	16.0	4.9		10.7 ± 2.2
2106	16.1	5.8		10.4 ± 4.1
1102	16.7	4.8		11.8 ± 2.7
7102	16.6	4.8	37.2 ± 4.7	
3205	18.2	8.1	8.9 ± 2.2	
6310	26.0	7.4		4.0 ± 1.4
302	26.1	6.7		1.8 ± 0.8
5402	28.3	3.7		1.8 ± 1.0
4401(BSP5)	28.6	3.5	15.6 ± 4.0^a^	2.2 ± 0.7^b^
6705	61.8	5.2		1.5 ± 0.5
1705	62.4	5.3		2.5 ± 1.1
3802(ALBUM)	63.3	5.4		1.8 ± 0.4

*Notes*: Protein spots numbers refers to those depicted in Fig. 3. Rainy season refer to Breeding Season 1 and Rest Period 1, while dry season refer to Breeding Season 2 and Rest Period 2, according to Fig. 2. MW and pI are the molecular weight and the isoelectric point of seminal plasma proteins from Brahman bulls. Averages followed by different

a,b superscripts are significantly different (*p* < 0.05) by the F-test in the ANOVA procedure.

**Table 6:** Concentration of SPP associated with fertility, differentially expressed in the most fertile bulls compared to the less fertile bulls of high and low fertility.

Protein spots^a^	Molecular weight/Isoelectric point	Protein concentration ± SD^b^	

		High fertility bulls	Bulls low fertility
2006, 5102 (Z13)	13–16 kDa/pI 5.5– 6.7	5.5 ± 0.0	27.4 ± 0.7
8304, 8305 (PROSTAGDSIN)	26 kDa/pI 6.2	34.9 ± 0.4	6.9 ± 0.4
5401, 402 (BSP5)	28–30 kDa/pI 3.6–5.2	1.8 ± 0.4	9.3 ± 0.7
3802, 3804 (ALB)	63–64 kDa/pI 5.7–6.5	21.3 ± 0.7	28.9 ± 1.1

*Notes*.

a assigned by the PD QUEST programme (Bio-Rad);

b standard deviation; They presented statistical differences (*p* < 0.05) between the two groups by the Tukey’s test.

**Table 7:** SPP concentration frequently ≥ 70% in high fertility bulls and low fertility bulls in the rainy season.

Protein spots^a^ (Molecular weight/Isoelectric point)	Protein concentration ± SD^b^	

	High fertility bulls	Bulls low fertility
1004 (14.0 kDa/4.8)	5.3 ± 3.3	5.2 ± 3.4
2005 (14.1 kDa/4.9)(BSP1)	4.2 ± 1.7	6.0 ± 2.6
3103 (16.0 kDa/4.9)(BSP3)	6.2 ± 3.3^c^	
2105 (16.2 kDa/4.8)	9.9 ± 3.0	2.8 ± 0.6
7102 (16.6 kDa/4.8)	13.1 ± 2.9	9.3 ± 4.6
1102 (16.7 kDa/4.8)	5.7 ± 3.4^c^	
7301 (17.1 kDa/9.2)	2.0 ± 1.0	3.5 ± 3.1
3205 (18.2 kDa/8.1)		4.1 ± 2.6^c^
4401 (28.6 kDa/3.5)(BSP5)		3.1 ± 1.2^c^

*Nota*.

a assigned by the PD QUEST programme (Bio-Rad);

b standard deviation;

c presented statistical differences (*p* < 0.05) between the two groups of males.

**Table 8:** SPP Concentration with frequency ≥ 70% in high fertility bulls and low fertility bulls in dry season.

Protein spots^a^ (MW/pI)	Protein concentration ± SD^b^	

	High fertility bulls	Bulls low fertility
9001 (13.4 kDa/9.7)		4.5 ± 3.0^c^
1004 (14.0 kDa/4.8)	4.1 ± 2.2	3.0 ± 1.8
0001 (14.0 kDa/4.4)	2.0 ± 0.4^c^	
3002 (14.1 kDa/5.3)		0.6 ± 0.3
2005 (14.1 kDa/4.9)(BSP1)	2.3 ± 0.0	2.5 ± 2.0
2006 (14.8 kDa/5.4)(Z13)		1.8 ± 0.5^c^
0002 (15.5 kDa/6.6)(BSP1)		0.4 ± 0.1
5102 (15.6 kDa/6.4)(Z13)		0.7 ± 0.1^c^
5105 (15.7 kDa/4.9)		6.0 ± 2.4^c^
1104 (15.7 kDa/5.5)		1.8 ± 1.1
3103 (16.0 kDa/4.9)(BSP3)	3.2 ± 2.2	5.4 ± 2.8
2106 (16.1 kDa/5.8)	2.3 ± 1.6	3.9 ± 2.8
7102 (16.6 kDa/4.8)	6.4 ± 3.0^c^	
1102 (16.7 kDa/4.8)	4.0 ± 0.8	5.1 ± 2.6
4401 (28.6 kDa/3.5)(BSP5)		1.8 ± 0.7
6705 (61.8 kDa/5.2)		2.2 ± 0.2^c^
3802 (63.3 kDa/5.4)(ALB)		3.0 ± 0.3^c^
2801 (63.5 kDa/5.4)		1.8 ± 0.6^c^
3804 (63.5 kDa/5.8)(ALB)		0.7 ± 0.1

*Notes*.

a assigned by the PD QUEST program (Bio-Rad);

b Standard deviation;

c presented statistical differences (*p* < 0.05) between the two groups of males.
